# 

**Published:** 1865

**Authors:** 


### NOTICE.

The Indiana State Dental Association will hold its next annual meeting at Indianapolis, on the last Tuesday of June, 1865.

A full attendance of all interested in the welfare of the profession is earnestly desired.

JOS. RICHARDSON, Cor. Sec.

### GOING ABROAD.

Our esteemed friend and Professional Brother, Dr. G. A. McClelland, of Louisville, Ky., within a few days starts on a tour to Europe for the double purpose of observation and invigoration. We hope and wish for him the entire fulfillment of his most sanguine anticipations. It gives us great pleasure to say to members of our profession abroad, that in Dr. M. they will find high professional attainments, with a full and just conception of the status and requirements of our profession, and a to will labor for its progress and welfare.

It gives us great pleasure to commend him to the kind regard of our professional brethren across the water.

### CONTENTS.

### The Dental Register

OF THE WEST,

*Issued Monthly, at $3 a Year in Advance.*

DEVOTED TO THE INTERESTS OF THE DENTAL PROFESSION.

**EDITED BY J. TAFT**

The volume commences in January and closes in December.

The Register being issued monthly is always fresh, therefore indispensable to the practitioner who desires to keep posted up with the new inventions and improvements which are daily developed. Neither expense nor effort will be spared to give value and variety to its contents. Articles will be illustrated with well executed engravings whenever they will add to the interest or assist in explanation.

Its extensive circulation and frequency of issue makes it one of the BEST DENTAL ADVERTISING MEDIUMS in the United States.

RATES OF ADVERTISING.

One page, 1 year, $50. Half page, 1 year, $30. Quarter page, or card, 1 year $20.

All advertising bills payable in advance, or at furthest after the issue of the first number ontaining the advertisement. All transient advertisements to be paid for in advance.

☞ CONTRIBUTIONS SOLICITED.

Address, J. TAFT, or "DENTAL REGISTER," Cincinnati, Ohio.

Business Communications to

**J. TAFT, Publisher,**
No. 172 Race Street.

